# The Australasian Virtual Herbarium: Tracking data usage and benefits for biological collections

**DOI:** 10.1002/aps3.1026

**Published:** 2018-03-02

**Authors:** David J. Cantrill

**Affiliations:** ^1^ Royal Botanic Gardens Victoria Private Bag 2000, Birdwood Avenue South Yarra Victoria 3141 Australia

**Keywords:** bioinformatics, data usage, herbarium records

## Abstract

**Premise of the Study:**

Globally, natural history collections are focused on digitizing specimens and information and making these data accessible. Usage information on National Herbarium of Victoria data made available through the Atlas of Living Australia and The Australasian Virtual Herbarium (AVH) is analyzed to understand how and by whom herbarium data are being used.

**Methods:**

Since 2010, AVH data usage information has been gathered from users and supplied to data custodians as a spreadsheet that includes number of download events, number of records downloaded, and user reasons for downloading data in predefined categories.

**Results:**

Since 2010, in excess of 268,000 download events of 194 million records (excluding testing events) have been recorded for the National Herbarium of Victoria data set. This means, on average, every record has been downloaded 220 times in the past nine years. Data use grew continuously from 2010 to 2015 but decreased in 2016 due to fewer ecological projects.

**Discussion:**

Data have primarily been used for ecological research, but there is an emerging trend for use in education including citizen science projects. Information about data use demonstrates relevance to funding agencies and helps inform the development of collections and prioritization of resources when digitizing material.

The origin of herbaria can be traced back to the Italian botanist Luca Ghini in the mid‐1500s, who developed and promoted the practice of pressing plants into herbaria (Arber, 1938). These early herbaria were mounted in books and use was closely tied to herbals. Over time, the nature of herbaria has changed from bound books of pressed plants used by individuals to loose sheets housed in cabinets. Both the size and number of collections have grown, and today approximately 3000 herbaria and more than 380 million specimens are housed globally in these collections (Thiers, 2017). These collections are a vast resource for the botanical research community but until recently have been neither easily accessible nor linked. Digitization of information about the specimens and the specimens themselves is rapidly changing this situation.

The use of herbaria has also changed over time from the initial role in herbal medicine and plant identification through to today where more than 72 uses have been documented (Funk, 2004). Recent innovative uses have included: demonstrating that herbarium collections document plant responses to historical changes in carbon dioxide (e.g., Woodward, 1987), tracking isotopic signatures of environmental change (e.g., Pedicino et al., 2002), and extracting DNA for analysis (e.g., Staats et al., 2011) to name but a few. All of these examples rely on access to the physical specimens; however, digitization opens up new possibilities including access to large data sets (Soltis, 2017). Aggregated data from many herbaria provide large spatial‐ and temporal‐scale data sets that have been used, for example, to model plant distributions and assess the impact of climate change (e.g., González‐Orozco et al., 2016). The changes in use over the past 500 years raises the question: What new uses will herbarium specimens and, in particular, digitized material be put to in the future?

Herbaria specimens document the occurrence of species at a particular place and a specific point in time. Unlike observational data, and because they are backed up by physical specimens, these data points can be updated as taxonomic concepts change. Each generation of scientists adds to these vast data repositories, improving them for the next generation. Indeed, it could be argued that, as scientific infrastructure, herbaria are one of the few that actually get better with time as knowledge attached to the specimens is gathered and improved. These attributes, together with the length of time that herbaria have been assembled, mean that herbaria represent one of the best‐quality temporal and spatial data sets we have of biodiversity.

In the 1980s, the herbarium community began capturing information about specimens into in‐house databases, and ultimately these were made accessible through the World Wide Web. Large‐scale efforts to make information housed in natural history collections accessible have been the focus of recent investment in digitization at both national (e.g., Australasian Virtual Herbarium, iDigBio) and international (e.g., Global Biodiversity Information Facility) scales. More than 25 years ago, the Australian plant taxonomic community identified the opportunity that digitization of collection information offered to improve collection management and streamline taxonomic processes. In 2000 a pilot project at the Royal Botanic Gardens Victoria was followed by an initiative led by the Council of Heads of Australasian Herbaria (CHAH), which resulted in the creation of Australia's Virtual Herbarium in 2001. This Australia‐wide resource containing specimen information from all the major state collections was developed between 2001 and 2006. The success of Australia's Virtual Herbarium ultimately led to funding for the Atlas of Living Australia (ALA). The ALA provided an opportunity to both upgrade and, more recently, integrate the data with New Zealand's Virtual Herbarium to create The Australasian Virtual Herbarium (AVH).

Understanding usage of AVH initially relied on web statistics (e.g., page views), which demonstrated the volume of use but gave little information on how the data were being used. Before 2010, use cases were also collected from AVH users who wanted access to large data sets. This was achieved by setting a download limit (10,000 records) that then required users to email and request access to the data. Through this method, individual use cases, such as predicting the invasiveness of Australian *Acacia* outside its native range (Hui et al., 2011), were gathered. However, these individual use cases gave no insight into the majority of AVH use. The creation of the ALA and the redevelopment of the AVH interface enabled a simple monitoring tool to be built that reduced the reliance on web statistics. Before a user can download any data query, they are required to answer some questions about how those data will be used, and this information is gathered automatically and provided back to data custodians. In this study, I present results on the use of Royal Botanic Gardens Victoria data (National Herbarium of Victoria [MEL] data set) extracted by users through the AVH interface. The uses are classified into several categories defined by the ALA.

## Methods

Records from the Royal Botanic Gardens Victoria are continuously contributed to the AVH and so the pool of data available for queries has grown from 791,106 records in 2009 to 881,328 records by June 2017. These small incremental increases are not considered to impact the data use requests. Queries against the AVH data set are provided by the ALA through the Collectory pages (Atlas of Living Australia, 2017a) on each individual institution's home page under the “download usage stats” button (Atlas of Living Australia, 2017b). These data are collected as part of the conditions of download. whereby users who wish to download data sets are required to provide a reason for the download from a menu of options (Table 1). Once the rationale for the download is provided, the data are emailed to the user, and the query recorded and added to the spreadsheet under the relevant data use category.

**Table 1:** Number of download events between October 2010 and July 2017 separated into download categories for data provided by the Royal Botanic Gardens to The Australasian Virtual Herbarium. Note that not all cells are filled because data use categories were added and deleted during the period considered

Category	No. of download events							
	2010	2011	2012	2013	2014	2015	2016	2017
Biosecurity			79	129	58	63	215	164
Citizen science							388	311
Collection management			33	62	83	83	112	66
Conservation management			502	1057	769	160		
Ecological research			1521	8738	20,609	87,043	8279	54,237
Education			226	513	806	704	995	742
Environmental assessment			271	500	355	403	950	520
Other			199	191	297	208	446	887
Other scientific			13	132	87	105	205	23
Restoration/remediation							79	46
Scientific research			1141	11,902	19,544	23,683	5948	3877
Systematics/taxonomy			101	485	165	293	505	296
Testing			146	209	6226	6453	913	629
Unclassified	6	348	495	112		620	1101	403
Total	6	348	4727	24,030	48,999	119,818	20,136	62,201
Total: testing	6	348	4581	23,821	42,773	113,365	19,223	61,572

Data use statistics are provided to data custodians broken down on a monthly basis and include: number of download events (i.e., queries) and number of records downloaded; each of these is broken down by data category. Data use categories have changed over time. From October 2010 to February 2012, download events and records downloaded were recorded in one general use category (“unclassified”). In March 2012, seven categories were added (“collection management,” “scientific research,” “education,” “environmental assessment,” “biosecurity,” “conservation management,” “other”). In April 2012, three further categories were added (“ecological research,” “systematics/taxonomy,” “testing”), and in May 2012 the category “other scientific research” was added. These categories remained stable until May 2016 when two more categories were added (i.e., “citizen science,” “restoration/remediation”). In total, 12 categories are currently available but 14 categories of data use are being reported to data providers. This appears to be due to old instances of the ALA still being available and to categories that are no longer in use (e.g., “unclassified”) in the current version still being recorded by the data logging system.

The addition and deletion of categories create some limitations for the study here, especially for comparison across years, where the atomization of categories will have undoubtedly impacted the use category a user would select when providing information. For example, increased atomization of data use should reduce the number of queries in the “other” category, as more choices are available to users. Other impacts are also likely. For example, the addition of the “citizen science” category in 2012 may have impacted the “education” category, the “scientific research” category, or the “other scientific” category. These impacts cannot be easily accounted for in any analysis through time. In order to minimize the impact of changes in data use categories, the following approach was taken. Data categories were aggregated into scientific research and non‐research uses. Non‐research use includes: “biosecurity management,” “conservation management,” “collection management,” “environmental assessment,” “citizen science,” “restoration/remediation,” “unclassified,” “other.” Research use includes: “scientific research,” “ecological research,” “systematics/taxonomy,” “other scientific research.” Testing events and records downloaded (Tables 1, 2) represent internal data use and so are discussed separately.

**Table 2:** Number of records downloaded between October 2010 and July 2017 separated into download categories for data provided by the Royal Botanic Gardens to The Australasian Virtual Herbarium. Note that not all cells are filled because data use categories were added and deleted during the period considered

Category	No. of records downloaded							
	2010	2011	2012	2013	2014	2015	2016	2017
Biosecurity			8955	776,223	5,473,629	6477	61,469	46,454
Citizen science							215,209	191,432
Collection management			24,484	138,421	697,538	153,553	791,900	2,276,059
Conservation management			256,092	2,420,607	9,422,268	103,650		
Ecological research			1,047,310	4,211,849	11,426,007	18,989,229	7,306,446	8,598,071
Education			117,264	638,298	8,228,089	953,809	5,835,867	476,086
Environmental assessment			67,088	338,508	466,260	217,612	1,278,404	290,937
Other			299,480	82,853	3,651,226	238,486	688,509	967,554
Other scientific			75,690	92,465	882,619	274,805	1,027,189	80,519
Restoration/remediation							13,000	3490
Scientific research			2,189,368	9,996,506	16,844,505	14,043,496	9,444,494	7,461,793
Systematics/taxonomy			27,938	643,602	788,519	215,871	111,025	124,440
Testing			444,812	521,139	514,455,610	187,502,064	7,276,899	1,061,741
Unclassified	8720	48,972	1,284,405	151,981	0	15,311,870	8,114,798	2,693,587
Total	8720	48,972	5,842,886	20,012,452	572,336,270	238,010,922	42,165,209	24,272,163
Total: testing	8720	48,972	5,398,074	19,491,313	57,880,660	50,508,858	34,888,310	23,210,422

Although the data use is captured on a monthly basis, for this study it has been consolidated into an annualized total of queries (download events; Table 1) and records downloaded (Table 2). Data were aggregated in non‐research use and scientific research use and are reported as annualized frequencies (Tables 1, 2). Monthly frequency data for ecological research are also presented as an aggregated monthly mean across the 2012 to 2016 period to illustrate the annual pattern of use. For 2017, data were extracted at the end of June and numbers have been doubled to give an estimated annualized total.

## Results

### Testing

The robustness of the search interface is tested through the external download function through a series of standard searches. These protocols ensure that new data uploads have happened correctly and that data supplied by the data custodians are mapped to the correct fields in the AVH cache. Data downloaded for testing are given in Tables 1 and 2 but are excluded from further analysis as these data represent internal use by the data custodians. In 2014 and 2015, major system upgrades to the Royal Botanic Gardens Victoria collection management system and the AVH system occurred. As part of these upgrades, additional testing events were run to check that both the data migration to the new collection management system and the redeveloped AVH portal were returning correct search results. Consequently, large peaks in testing events are observed in 2014 and 2015.

### General data use

**Figure 1:**
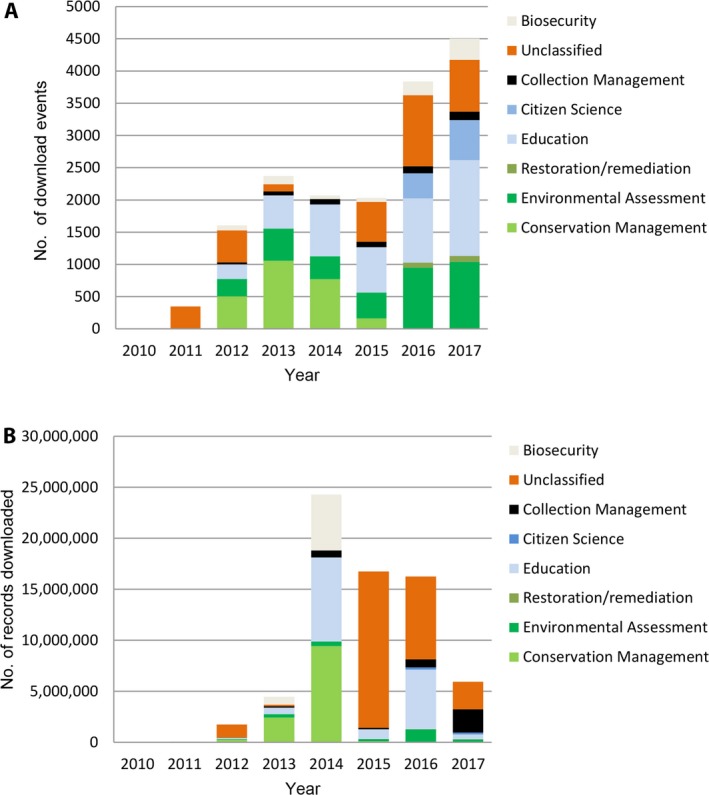
Non‐research data use presented as download events (A) and records downloaded (B). Note 2017 data only represents January to July.

Overall queries increased from 2011 to 2013, declined in 2014 and 2015, and have increased again in 2016 and 2017 to more than 4500 per year (Fig. 1A). This contrasts with the number of records downloaded, which peaked in 2014 (Fig. 1B).

### Non‐research use

This includes uses in the “biosecurity,” outreach (“education” and “citizen science”), “conservation management,” and “collection management” categories. The number of download events increased over the seven‐year period, although with a slight decline in 2014 and 2015 (Fig. 1A). This contrasts with the number of records downloaded, which peaked in 2014 (Fig. 1B).

### Scientific research use

**Figure 2:**
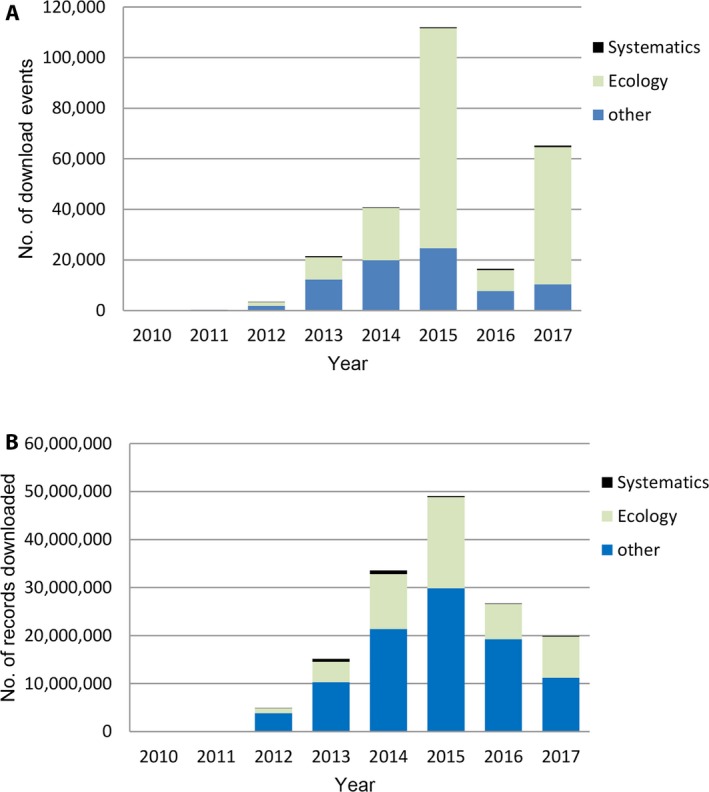
Scientific research data use presented as download events (A) and records downloaded (B). Note 2017 data only represents January to July.

Use for research rose to a peak in 2015 when a large number of small queries for ecological research occurred. A dramatic fall in queries occurred in 2016, but this decrease has been reversed in 2017 with projected number of queries to exceed 2015 (Fig. 2A). Similar to the queries, the number of records downloaded peaked in 2015, declined in 2016 (but not as markedly as the queries), and is projected to recover in 2017 but not to 2015 levels (Fig. 2B).

## Discussion

Prior to October 2010, our understanding of AVH data use was restricted to requests for large data sets and web statistics on page views. The AVH had a limit on the number of records that could be downloaded (10,000). If users needed data sets larger than the data download limit, they were required to contact an administrator to request permission. By designing smaller queries, users could get around this feature and so extract large data sets. Requests for large data sets highlighted the value placed on the data that were being made available but was largely ad hoc, resulting in a limited understanding of how the data were being used more broadly (e.g., Saintilan, 2009; Waters et al., 2010; Hui et al., 2011). The use cases for large data sets obtained through this process allowed data custodians to demonstrate the impact of making the herbarium specimen data free and easily accessible. They were important for demonstrating value when seeking further financial support to build on the original AVH. However, they only captured a small subset of the total data usage. The advantage of this manual approach to understanding how herbarium data were being used was that the impact of the data use could be demonstrated. However, it came with a time and administrative cost.

The creation of the ALA allowed further development of AVH, including expanding it to cover Australasian herbaria as well as implementation of the data use system that is reported here. The data use system is automated, resulting in a lower administrative burden but at the cost of information richness, particularly the impact of the data use. The use data were broken down into two main groups, scientific research and non‐research uses.

### Non‐research uses

In 2014, a large peak in records downloaded was seen, which has been interpreted as users taking the whole data set and loading it into local systems to reduce the need to repeat queries in the AVH, or for other analysis that required data modifications. Recent publications confirm this approach by the research community (e.g., Dodd et al., 2015, 2016). Large data downloads may mask underlying trends in data use that will become more evident with time. Collection management was a small component of data use, being under 1% of the download events between 2012 and 2017. However, the number of records downloaded has increased as a percentage of downloads from 0.45% in 2012 to 9.8% in 2017. This suggests that queries of the whole AVH data set offer some benefit for collection management and may relate to an increase in data‐cleaning activity by the community.

**Figure 3:**
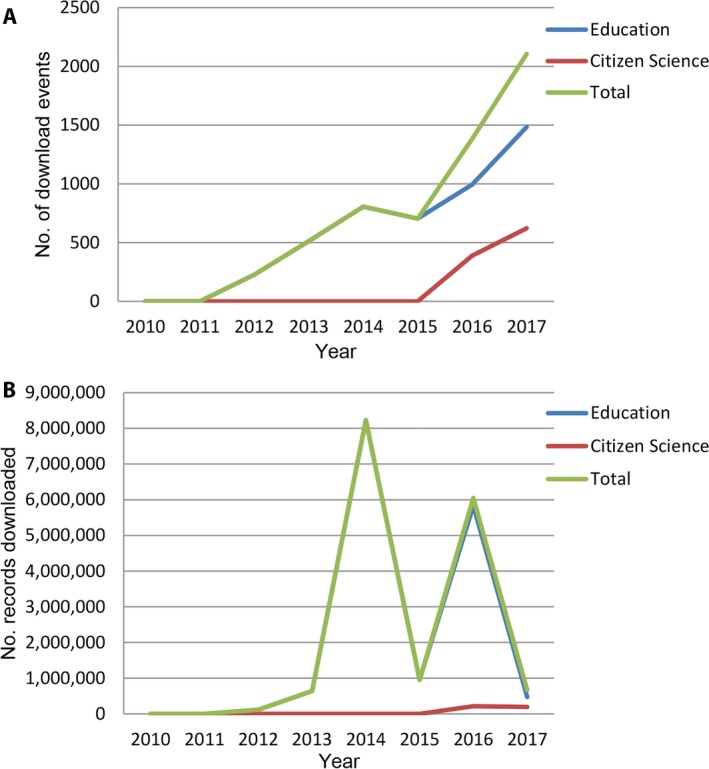
Outreach uses as download events (A) and records downloaded (B).

Uses in the categories “education” and, more recently, “citizen science” can be viewed as outreach activity. Download events in these categories have grown from 226 in 2012 to 1383 in 2016 and are projected to exceed 2000 in 2017 (Fig. 3A). This contrasts with the number of records downloaded, which has fluctuated from year to year with peaks in 2014 and 2016 (Fig. 3B). The overall trend is for a decrease in the size of downloads, suggesting that users are becoming more discriminating in the data extracted.

**Figure 4:**
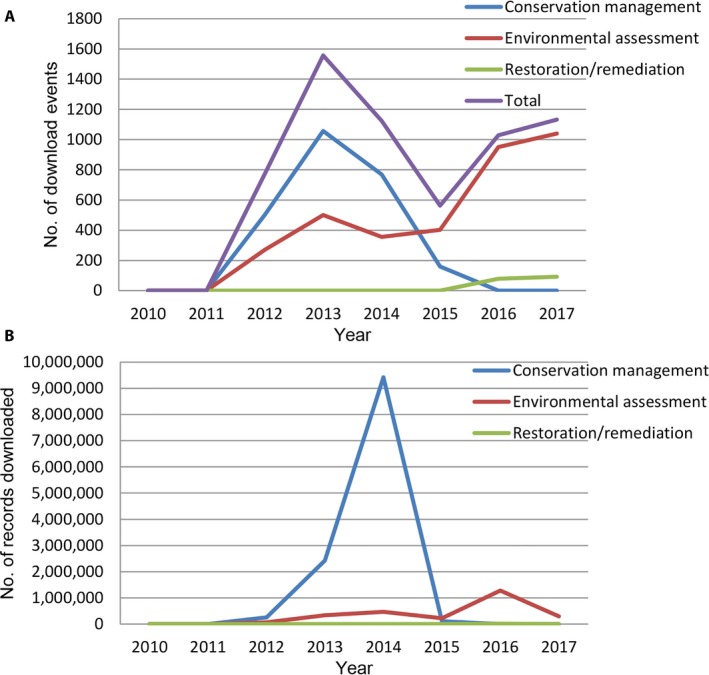
Conservation uses as download events (A) and records downloaded (B).

The categories “conservation management,” “environmental assessment,” and “restoration/remediation” can be viewed as conservation activity. Download events in these categories rose to a peak in 2013 and declined in 2014 and 2015 before rising again (Fig. 4A). This contrasts with the records downloaded, which comprised some very large downloads in 2013 and 2014 (Fig. 4B). The size of these downloads suggests that some users downloaded the whole data set, likely to populate in‐house database systems (e.g., AVH or subsets of AVH are part of the Australian National Heritage Assessment Tool [ANHAT] Database; Laity et al., 2015; Burley et al., 2016a, 2016b). The decline in use from 2013 coincides with the downturn in mining activity and development of new mines, therefore reducing the regulatory requirements for conservation assessments. This interpretation suggests the AVH data are being used to inform planning for new developments.

### Scientific research

Despite the importance of herbarium specimens as vouchers for systematic and taxonomic research, data use by this research group forms a small percentage of the total use, ranging from 0.39% to 2.63% of download events and 0.32% to 3.30% of the records downloaded in any year (Fig. 2A, B). This contrasts with ecologists, who are the major users of AVH data. Downloads for ecological research peaked in 2015 at 87,043 queries and nearly 19 million records (average 218 records per query) (Tables 1, 2). This contrasts with use in other years where there were fewer queries of a larger size. For example, in 2016 there were 8279 query events extracting 7.3 million records (average 882 records per query). The peak use in 2015 suggests a large project with multiple small queries, perhaps for species niche modeling. From the published literature, we can identify studies that have taken large downloads and then modified (or removed) records for analysis, such as the study of plant invasions and naturalizations in Australia (Dodd et al., 2015, 2016). The combination of literature searches and the download statistics gives us some insights into herbarium data use.

**Figure 5:**
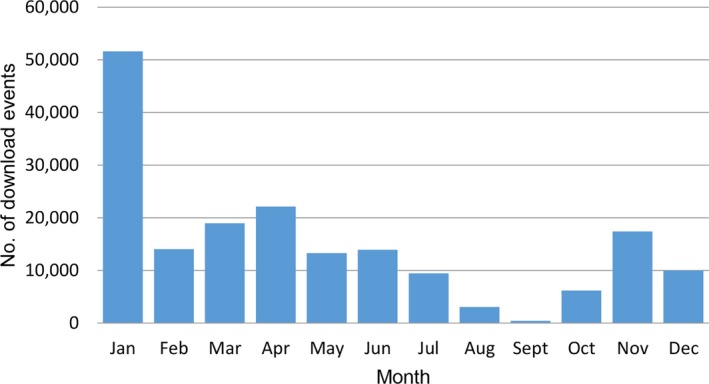
Monthly use of Royal Botanic Gardens Victoria data for ecological research based on download events.

Other patterns of use are apparent when the data for ecological research are examined on a monthly basis (Fig. 5). Here, data queries peak in January, coinciding with the major break in academic teaching (December to March) and grant preparation for submission in March. There is also a marked lack of use in August and September, probably coinciding with the main period of fieldwork in spring. This usage pattern could be used to inform decisions about when to upgrade the system in order to minimize disruption to users.

### Data use and recommendations

The manual approach (2001 to 2006) to data use reveals the AVH data were being used for a diversity of purposes, including the generation of species lists for large areas to aid assessment in planning for new national parks and also for World Heritage applications. Other data uses included species niche modeling for Australian taxa that had become weedy overseas (e.g., *Acacia* species; Hui et al., 2011), for which the climate envelope occupied by the taxon in Australia was used to predict the potential range in the country where they had become weedy. This information was used to prioritize taxa for eradication and target areas for control to reduce the projected impact of the target taxa. Although the manual method provided a rich source of the impact of AVH data, it was limited to a very small set of data uses.

In contrast, the automated tool provides a useful overview of all data uses by predefined category. This approach has limitations because it assumes the data custodians understand all of the use cases and can generate meaningful predefined categories, which may not be the case. On average, around one third of the queries (8.4% to 51.7%; average 34.82%) and records downloaded (9.2% to 71.00%; average 37.7%) are allocated to general categories (“other,” “unclassified,” “scientific research”). This limits our understanding of the research fields utilizing the resource and suggests that the use categories set by the data custodians need refinement. Alternatively, a different methodology is needed to better capture information on the purposes that users are downloading data and the user audience. Options may include further refinement of and addition of user categories. Alternatively, another alternative may be using a free text field when the general categories are selected that can be analyzed using machine learning or natural language processing to classify the groups.

Although the automated data use system provides important insights into overall data use, we currently rely on additional methods, such as publication searches, to understand use cases and likely impact. Based on literature searches across a range of academic databases, we have added to the use cases collected prior to the implementation of the automated data use system. Taxon distribution maps are generated and published routinely for systematic and taxonomic (e.g., Wilkins and Whitlock, 2016 as just one of many examples) studies. The distribution information is used in a variety of ways, including phylogeographic studies (e.g., Barrett et al., 2014; Bayly et al., 2016); continental‐scale diversity patterns and visualization of species richness (Stevenson et al., 2012; Nagalingum et al., 2015), endemism (Mishler et al., 2014), and using these data to examine spatial patterns of species turnover (González‐Orozco et al., 2014a). The integration of distributional data with phylogenetic information has been used to generate patterns of phylogenetic diversity (González‐Orozco et al., 2016; Thornhill et al., 2016) and turnover (Laffan et al., 2016) and indices developed from these studies can be used to inform conservation policy (Laity et al., 2015). The development of measures of phylogenetic diversity and endemism has been used to understand ecology of rainforests and to inform conservation actions (e.g., Kooyman et al., 2013). AVH data are also being used as part of analyses to: understand spatial biases in biodiversity data sets (Haque et al., 2017); relate distribution patterns to physical variables such as soil and climate (Prentice et al., 2017); examine the spatial distribution of traits (Moles et al., 2014; Tindall et al., 2017); combine species distributions with phylogenetic and environmental variables to identify biogeographic regions and understand evolutionary patterns of plant distribution (e.g., González‐Orozco et al., 2013, 2014b), or to relate diversity to primary productivity (Burley et al., 2016a) and ecosystem function (Burley et al., 2016b). Herbarium data have been used to test invasion models (e.g., Taylor and Kumar, 2012), to identify invasion hotspots (O'Donnell et al., 2012), and have been used in tandem with genetics to identify introduction events and invasion history (Dormontt et al., 2014). These studies have also been used to examine threats to biodiversity under climate change using forward modeling of native (e.g., González‐Orozco et al., 2016) and introduced (O'Donnell et al., 2012) species.

AVH data are also being integrated into other products. AVH data are readily imported into the ALA for further analysis using a range of ALA tools. Other digital products, such as VicFlora, generate species distribution maps on the fly from AVH data as part of the species profile pages (VicFlora, 2018a). Checklists are also generated by querying the AVH data set with a spatial query and compiling all the taxa supported by herbarium records in that region (VicFlora, 2018b).

The study reported on here has examined the use of the Royal Botanic Gardens Victoria data housed in the AVH. An interesting question for the future is: How representative are the data use and use cases identified for the National Herbarium of Victoria data set? It should be considered that each herbarium is unique, with its own collecting history both in time and space. It is likely that most user queries are searching across the whole AVH data set and so are blind to the data providers (unless they filter on particular herbaria), which means that the pattern of queries and, therefore, data use is likely to be the same as that reported on here. However, individual herbaria are different sizes with different taxonomic and geographic coverage. For example, the Queensland Herbarium (BRI) only supplies records of Queensland taxa and so will not appear in any searches looking at non‐Queensland distributions. The Western Australian Herbarium (PERTH) collection is largely focused on Western Australian taxa and so will feature less prominently in studies of east coast taxa. Furthermore, legislation for environmental assessments has different requirements in different states; therefore, herbaria from different jurisdictions are likely to have slightly different query profiles in the conservation management area. The stringency of Western Australian environmental requirements means that this data set may well be used more extensively by consultants in Western Australia than other data sets. These are areas that remain to be examined and are beyond the scope of this study.

## Conclusions

Digitization and making data accessible from herbarium collections has opened up new opportunities that demonstrate the relevance of these resources for the wider scientific community. Herbaria are no longer specimen repositories used by a limited number of taxonomists; they are now rich data sources that have many applications. While specimens remain critical for fixing names and creating a stable taxonomic framework that underpins all of biology, the use of natural history data for other scientific endeavours is just beginning. It is critical for custodians of natural history collections to understand how data and digital objects are being used and how often. The data use function developed for the data custodians within the ALA gives important insights into data use and data use patterns. Nevertheless, this system could be improved given that approximately one third of all queries are not currently classified into specific use cases. Future implementation of these types of systems needs to address this gap to allow a more comprehensive understanding of data use to be gained. Although we broadly understand the data use, we do not have a good handle on the impact of these projects due to the general nature of the data collected. In order to demonstrate relevance, we need tools to understand both the totality of the use and, importantly, the impact of the use case on end users.

## Data accessibility

Data are accessible via the Atlas of Living Australia web portal to the Collectory pages found at (http://collections.ala.org.au/). The data set used in this study is available on the Royal Botanic Gardens Victoria page (http://collections.ala.org.au/public/show/in21) under the National Herbarium of Victoria data set (http://collections.ala.org.au/public/show/co55) and can be freely downloaded by clicking on the “download usage stats” button.
